# Methylenetetrahydrofolate Reductase (MTHFR) Variants and Severe Capecitabine Toxicity: A Case Report and Review of Literature

**DOI:** 10.7759/cureus.75791

**Published:** 2024-12-16

**Authors:** Li Lingyan, Wang Linjun, Zhong Wenjun

**Affiliations:** 1 Oncology, Qiannan People's Hospital, Duyun, CHN

**Keywords:** 5-fluorouracil, capecitabine, colorectal cancer, dihydropyrimidine dehydrogenase, methylenetetrahydrofolate reductase, toxicity

## Abstract

Capecitabine is an oral prodrug metabolized into 5-fluorouracil (5-FU) and serves as a representative anticancer agent. While fluoropyrimidine treatment is usually well-tolerated, a subset of patients unfortunately experiences severe and sometimes life-threatening toxicity related to these compounds. This adverse reaction is frequently attributed to partial or complete deficiencies in the dihydropyrimidine dehydrogenase (DPD) enzyme. However, some patients may still suffer from severe toxic effects despite normal DPD screening results when treated with capecitabine. This paper presents the case of a Chinese woman with stage IIIB moderately differentiated adenocarcinoma of the lower rectum (cT3N2aM0) who exhibited severe toxicity after two weeks of neoadjuvant concurrent chemoradiotherapy in TNT mode at a low dose (825mg/m2 bid) of capecitabine. We found that this severe toxicity might be attributable to insufficient methylenetetrahydrofolate reductase (MTHFR) activity. To our knowledge, such reports are scarce in the medical literature concerning the Chinese population.

## Introduction

Fluorouracil anticancer drugs, particularly 5-fluorouracil (5-FU) and capecitabine, well serve for treating various cancers, including breast cancer (BR), colorectal cancer (CRC), head and neck cancer (HNC) and gastric cancer (GC). Each year, two million patients receive fluoropyrimidine treatments which are generally well-tolerated, however, the associated toxicity is significant, affecting about 30% of patients, with 0.5%-1% experiencing fatal outcomes [1]. Dihydropyrimidine dehydrogenase (DPD) critically participates in metabolizing 5-FU and capecitabine, accounting for approximately 80% of 5-FU catabolism. Over the past three decades, clinical evidence has consistently shown that DPD deficiency is obviously linked to severe and lethal fluoropyrimidine toxicity, which results from deleterious polymorphisms in the gene encoding DPD (DPYD). Known DPYD gene variants such as DPYD*2A, c.2846A>T, c.1679T>G, and c.1129-5923C>G (HapB3) are linked to increased 5-FU-related toxicity in multiple studies [2]. Some experts contend that DPD polymorphism is not the sole factor influencing drug sensitivity; other genetic polymorphisms may also impact drug metabolism and toxicity [3]. Our literature review revealed that polymorphisms in genes such as thymidylate synthase (TYMS) and methylene tetrahydrofolate reductase (MTHFR) could contribute to toxicity in a similar manner as DPYD variants [4]. However, data on MTHFR remain sparse. We report a case of a female patient from China with stage III rectal cancer who experienced severe toxicity during neoadjuvant concurrent chemoradiotherapy in TNT mode with lower-than-usual doses of capecitabine (a precursor to 5-FU). Our testing found that several of her common DPYD gene mutation sites sequenced normally, MTHFR (677C>T) heterozygous mutations.

## Case presentation

**Figure 1:**
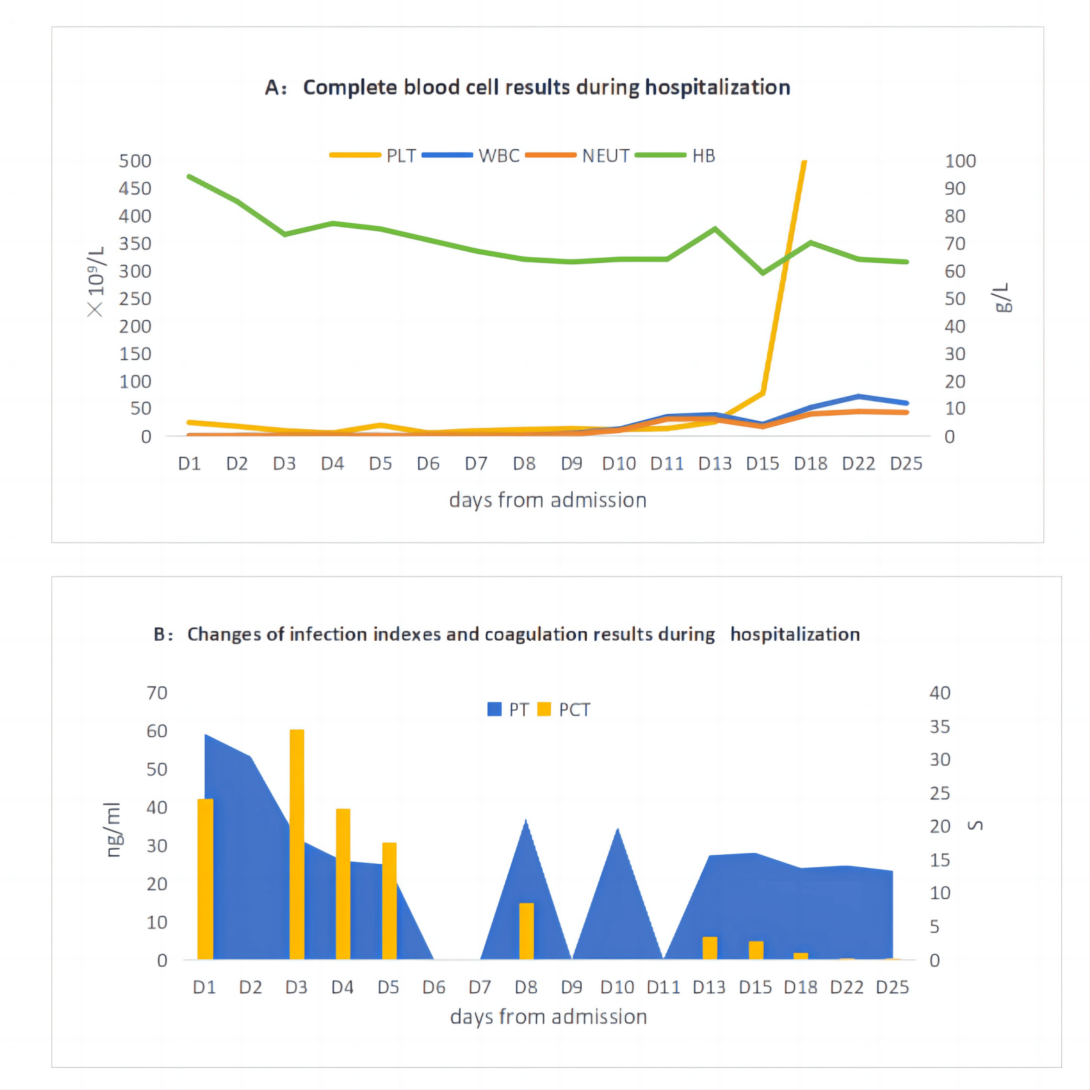
These two charts illustrate the changes in the complete blood count, coagulation index, and infection index of the patient during hospitalization. PLT: blood platelet; HB: hemoglobin; WBC: white blood cell; NEUT: neutrophil granulocyte; PT: prothrombin time; PCT: procalcitonin

A female patient in her 60s (previously in good physical condition, no history of special diseases and family history), initially self-treating hemorrhoids due to "intermittent blood in the stool for eight months," did not observe improvement and subsequently visited a local hospital. A colonoscopy revealed periannular swelling protruding into the intestinal lumen at the rectum (2cm from the anal margin), causing luminal stenosis. The tumor surface exhibited congestion, edema, and erosion. A biopsy demonstrated brittle and easily bleeding tissue, and pathology confirmed moderately differentiated adenocarcinoma of the rectum. She was later admitted to our hospital, where rectal magnetic resonance imaging (MRI) revealed a thickening of the ring circumference in the middle and lower parts of the rectum, 4.6cm from the anal margin, with a thickness of about 1.0cm over a length of 4.3cm. Several enlarged lymph nodes in the mesangial area around the rectum and along the upper rectal artery were identified, numbering more than four, suggesting metastasis; some were adjacent to the mesenteric fascia. Diagnosis: middle and lower segment rectal carcinoma (clinical TNM stage: cT3N2M0 IIIB), MRF (mesorectal fascia) (+), EMVI (extramural vascular invasion) (+). Chest and abdominal pelvic CT (computed tomography) scans showed no distant organ metastasis. Based on these findings, the patient was diagnosed with stage IIIB (cT3N2aM0) intermediate differentiated adenocarcinoma of the lower segment rectal. Per guidelines, she was indicated for neoadjuvant concurrent chemoradiotherapy, excluding any treatment contraindications, and commenced radiotherapy. The radiotherapy schedule included: Gross tumor volume (GTV) encompassing the primary rectal lesion and metastatic lymph nodes in the mesenteric region and the upper rectal artery region, expanded by 5mm to form pathologically gross tumor volume (PGTV), with 95% PGTV dose at 50.6 Gy/22F. Clinical target volume (CTV) covered the primary rectum lesion and its upper and lower 2cm, the internal iliac, obturator, anterior sacral lymph node drainage areas, and the mesorectal region, expanded by 5mm to form planning tumor volume (PTV), with 95% PTV dose at 41.8Gy/22F. Oral capecitabine chemotherapy was administered simultaneously, with the patient standing 168cm tall and weighing 70kg, her calculated body surface area was 1.81m^2^, dosing 825mg/m^2^, 1.5g p.o. bid d1-5/w. After the eighth radiotherapy session and eight days of oral capecitabine chemotherapy, a routine blood reexamination revealed a grade II decrease in white blood cells and neutrophils (leukocytes: 2.5×10^9^/L, neutrophils: 1.44×10^9^/L), and medications, such as Diyu Shengbai Pian and granulocyte-stimulating factor injection, were administered to elevate white blood cells. Chemoradiotherapy continued. After completing 13 sessions of radiotherapy and 13 days of oral capecitabine chemotherapy, the patient developed severe diarrhea, with watery stool over 10 times/day, approximately 100ml/day of blood in the stool, fever peaking at 39.3℃, and severe oral ulceration, leading to an inability to eat normally, with mild nausea and vomiting, and a cough producing white phlegm, and no numbness or pain in her hands and feet. The patient appeared extremely listless and in a poor mental state. She was promptly hospitalized; blood tests indicated severe grade IV myelosuppression (white blood cells: 0.07×10^9^/L, neutrophils: 0×10^9^/L, red blood cells: 3.16×10^9^/L, hemoglobin: 94.0g/L, platelets: 24×10^9^/L), and abnormal coagulation function (PT 33.8s). Procalcitonin (PCT) was abnormally elevated (42.13ng/ml). Given the critical state of her health, she received active symptomatic support treatment including leukocyte and platelet enhancement, anti-infection, hemostasis, diarrhea prevention, cooling, fluid replenishment, nutrition, and oral gargling. However, her condition showed little improvement, primarily due to difficult-to-correct bone marrow suppression (pancytopenia), with progressively declining platelets and hemoglobin, and ineffective red blood cell and platelet transfusions (refer to Figure 1 for changes in blood results during hospitalization).

Considering radiotherapy typically does not cause significant myelosuppression, the severe toxic reaction at a low dose of capecitabine was puzzling. Thus, we hypothesized the possibility of rare DPD enzyme deficiency causing intolerance to fluorouracil drugs. PCR (Polymerase Chain Reaction) testing of blood samples revealed that several common mutation sites of the DPYD gene in the patient were normal wild homozygous type (Table 1). The literature review indicated other genetic polymorphisms could cause similar toxicity. Examination of two common MTHFR genotypes found the patient's MTHFR (677C>T) CT was mutant heterozygous and MTHFR (A1298>C) AA was wild homozygous (Table 1).

**Table 1:** Whole-gene sequencing results for the DPYD and MTHFR genes. DPYD: Dihydropyrimidine dehydrogenase; MTHFR: Methylenetetrahydrofolate reductase.

Genetic test results			
Number	Detection site	Genotype	Result
1	DPYD*2A（476002G>A）	GG	wild homozygous type
2	DPYD*13（1679T>G）	TT	wild homozygous type
3	DPYD（2846T>A）	TT	wild homozygous type
4	MTHFR（1298A>C）	AA	wild homozygous type
5	MTHFR（677C>T）	CT	mutant heterozygous type

We considered that the patient's DPD enzyme activity level might be normal, but MTHFR enzyme activity was approximately 66% of normal, with insufficient folic acid utilization. Studies have shown that the response rate of CRC patients to the FOLFOX regimen with this enzyme activity is about 53.3%. Considering these enzyme activities, the patient's response to treatment with fluorouracil was moderate, but the risk of gastrointestinal reactions, mucositis, neutropenia, and hand-foot syndrome was increased. For the toxic side effects caused by low or lacking MTHFR enzyme activity, no specific rescue drugs are available, and active symptomatic supportive treatment remains the main approach. After nearly a month of active rescue treatment, the patient was finally cured and discharged from the hospital.

After discharge, the patient's anemia resolved through enhanced nutritional intake, resulting in no fatigue and discomfort, and his condition improved and stabilized. Upon returning to the hospital one month later, all clinical indicators had normalized, and a pelvic MRI evaluation indicated a reduction in the tumor in the middle and lower rectum. Surgical treatment was then recommended. Excluding surgical contraindications, the patient received laparoscopic radical resection of rectal cancer with ileostomy. Postoperative pathology demonstrated a moderate differentiation of adenocarcinoma, ypT3N1b, with two out of nine lymph nodes positive, TRG: Grade 1; no genetic tests were conducted. The patient recovered well postoperatively, and four cycles of adjuvant chemotherapy were recommended. Four weeks post-surgery, the patient commenced the first cycle of postoperative adjuvant chemotherapy. Specifics included: Height 168cm, body weight 65kg, body surface area 1.70m2, raltitrexed 3mg/m2 5mg d1+ oxaliplatin 130mg/m2 200mg d1, every three weeks (q3w). There were no symptoms of nausea, vomiting, abdominal pain, diarrhea, or other discomfort post-chemotherapy. Subsequent blood tests and liver and kidney function tests showed no significant abnormalities, except for mild anemia (Hb:102g/L), chemotherapy tolerance was good, with no serious adverse reactions previously noted.

## Discussion

5-fluorouracil (5-FU)-based therapy remains the primary chemotherapy method for colorectal cancer. Capecitabine, an oral chemotherapy drug, is metabolized into 5-FU in the body and is extensively used to treat various cancers. However, 19%-32% of capecitabine- and 5-FU-treated patients develop early-onset, severe, or lethal toxicity [5]. Notably, dihydropyrimidine dehydrogenase (DPD) crucially affects fluoropyrimidine inactivation, metabolizing over 80% of 5-FU in the liver. Patients with weakened DPD activity hold a larger likelihood of suffering severe fluoropyrimidine-related toxicity [2]. Substantial clinical evidence indicates that DPD deficiency or reduced activity slows the metabolism with regard to capecitabine, leading to an accumulation of 5-FU in the body and thus elevating toxicity risks. These toxicities may include gastrointestinal inflammation and ulcers, neutropenia, neurotoxicity, severe diarrhea, oral mucositis, hand-foot syndrome, dyspnea, and alopecia [6]. Since most 5-FU is degraded by DPD, the relevance of DPD deficiency to 5-FU-related severe toxicity is well elucidated in some studies [7]. Genetic defects in DPD activity are not uncommon in the population, and it is estimated that 9% of European patients have a variant of the DPYD gene capable of reducing enzyme activity, while about 0.5% of patients lack DPD altogether. However, the incidence of partial abnormality of DPD function is about 3%-5% in Caucasians, and only 0-0.7% in Asians [8]. Therefore, the large inter-individual difference in toxicity induced by fluoropyrimidine is partially caused by genetic factors. DPD activity is often linked to DPYD gene polymorphism. Many variants of the DPYD gene have been reported, and their functional significance is unclear. Known DPYD gene variants, such as rs3918290 (DPYD*2A), rs55886062 (DPYD*13), rs67376798 and rss56038477 (HapB3), can lead to stronger 5-FU-related toxicity in several studies [9]. According to the current clinical pharmacogenetic guidelines and medicine agency recommendations, four specific variants can be subjected to genotyping [10]. However, routine DPD enzyme testing is not recommended in China. Therefore, measuring DPD activity is not covered by public health insurance, and DPD activity is not tested in our daily clinical practice. At present, the drug labels of 5-FU and capecitabine do not include the adaptive administration strategies based on 5-FU polymorphism metabolism. However, due to our limited understanding of DPD detection, our institution only screens for three DPYD gene variants, potentially overlooking a crucial mutation and leaving uncertainty regarding the actual DPD status. This limitation highlights the need to accumulate case reports for further comprehension.

Methylenetetrahydrofolate reductase (MTHFR) is a key enzyme modulating folate metabolism and 5-FU cytotoxicity. MTHFR controls intracellular CH2FH4 concentration by irreversibly converting CH2FH4 to 5-methyltetrahydrofolate. An increase in the concentration of 5,10-methylenetetrahydrofolate is essential for the optimal effect of 5-FU. It is hypothesized that weakened MTHFR activity is possibly accompanied by a higher concentration of 5,10-MTHF, which in turn leads to cytotoxicity to fluoropyrimidine. In the MTHFR gene, several single nucleotide polymorphisms (SNPs) have been reported. According to previous reports, two common SNPs, C677T (rs1801133) and A1298C (rs1801131), are related to weakened enzyme activity, meanwhile changing intracellular folate distribution [11]. The C677T variant weakens the MTHF activity, causing a decrease of 30% and 70% in heterozygotes (CT) and homozygotes (TT), respectively. Also, the MTHFR A1298C polymorphism slightly weakens the catalytic activity of the enzyme, causing a decrease of 30%-40%. Heterozygote patients with both SNPs (677CT/1298AC) also exhibit synergically lower MTHFR activity. Reduced enzyme activity leads to elevated intracellular concentrations of 5,10-MTHF, which increases fluoropyrimidine toxicity. Our tests showed that the patient's MTHFR (677C>T) was a heterozygous mutation (CT), and MTHFR enzyme activity was approximately 66% of normal enzyme activity and decreased by approximately 34%. All these conform to previous studies. It is therefore reasonable to consider MTHFR gene variation as a possible cause of the severe toxicity in patients receiving low-dose capecitabine monotherapy. Genetic polymorphisms in MTHFR, particularly in MTHFR 677C>T and 1298A>C, can affect the chemotherapy efficacy and toxicity of fluorouracil drugs such as 5-fluorouracil (5-FU). Among the various studies examining the underlying predictive mechanism of C677T and A1298C gene variants against the toxicity as well as the efficacy of antifolate and fluorouracil drugs (methotrexate (MTX), 5-fluorouracil (5-FU), and raltitrexed) [12], some have revealed a close linkage between toxicity and MTHFR variants [13], while others have reported no association or opposite results [14, 15]. Significant interactions between MTHFR polymorphism and nutritional/environmental factors (folate status) and ethnicity have been reported [16, 17]. Also, the frequency of mutant alleles of the SNP varies by ethnic origin, with that for the MTFHR C677T mutant allele changing from 10% (in Asians) to 57% (in Mexicans Mestizos), and for the 1298 CC allele changing from 14% (in Asian) to 37% (in Caucasians) [11]. Due to such high variability, researches have not yielded consistent data with regard to the toxicity of fluoropyrimidine-based chemotherapy as well as its association with MTHFR polymorphism. Overall, a complex relationship exists between MTHFR gene polymorphism and 5-FU-based chemotherapy efficacy and toxicity in the treatment of CRC, which has many influencing factors, such as ethnic differences, chemotherapy regimen, individual folate levels, and environmental factors.

Thymidylate synthase (TS), an enzyme encoded by the thymidylate synthase gene (TYMS), crucially impacts the pyrimidine and DNA synthesis. TYMS is the enzyme that converts deoxyuridine monophosphate (d-UMP) to deoxythymidine monophosphate (d-TMP) to benefit DNA synthesis. The active metabolite of 5-FU with TYMS and 5,10-methylene tetrahydrofolate (5,10-mTHF) forms a complex, blocking the production of dTMP, resulting in reduced DNA synthesis, and d-UMP mistakenly incorporated DNA, causing DNA to break down. TYMS enzyme is one of the targets of 5-FU chemotherapy. The TYMS expression is relevant to the sensitivity to 5-FU, and despite the demonstration of this functional association with fluoropyrimidine metabolism and tumor response in various studies, the effect of genetic polymorphism on TYMS is unclear. Genetic polymorphisms in the five 'regulatory regions of the TS gene promoter, encompassing double (2R) or triple (3R) repeats of the 28 bp sequence, can affect TS expression [18]. TYMS promoter and 3'-UTR (untranslated region) ins/del genetic polymorphisms have been found to affect TS expression and are relevant to toxicity and improved clinical response [19]. Several studies have reported that pharmacogenetic variation of TYMS (TYMS 5 '-UTR VNTR and TYMS 3' -UTR 6-bp ins/del) is associated with increased toxicity and sensitivity to fluoropyrimidine [19-21]. Polymorphisms of TS (6 bp deletion at 3 'and 28 bp duplication, including G>C mutation at 5') were measured in 105 consecutive patients, showing that patients homozygous for the TS 3RG allele presented a higher global toxicity grade 3 and 4 versus those heterozygous for the 3RG allele or those without the 3RG allele (50% vs. 19% vs. 13% respectively, P=0.064)) [18]. Studies have also found that the 5'TSER genotype 2R/2R*f of TYMS polymorphism in patients is homozygous, which makes patients more sensitive to 5-FU drug and more susceptible to 5-FU toxicity [22]. According to previous studies, TSER 2R/2R genotypes present a close linkage with hematological and gastrointestinal toxicity [14]. However, other studies have produced conflicting results, and some scholars believe that the number of repeats in the TYMS 5 '-UTR region cannot impact the toxicity. Therefore, in meta-analyses, despite the relevance of TYMS 5 '-UTR region variations to adverse effects, their effects tend to be small and the test exhibits weaker feasibility [23]. Similarly, the impact of TS gene polymorphism in the 3 'region on toxicity is also contradictory [18, 19, 21], and its functional effects remain to be clarified by further studies.

Capecitabine is a prodrug of 5-FU, which first undergoes Carboxylesterase (CES) hydrolysis in the liver for the generation of 5-fluorodeoxycytidine, followed by the cytidine deaminase (CDA) deamination in liver and neoplastic tissue for the generation of 5-fluorodeoxyuridine. The generated 5-fluorodeoxyuridine is converted to 5-FU via thymidine phosphorylase (TP) [19]. SNPs or variations in the genes encoding these enzymes can affect the rate at which capecitabine is converted to 5-FU, thereby affecting its efficacy and toxicity [24]. In addition to DPYD, MTHFR, or TYMS, SNPs in CDA and CES are also associated with capecitabine efficacy and toxicity. The CDA gene exhibits a strong polymorphic property. Researches have not well interpreted the genotypes-phenotypes relationship. Upon the decrease in CDA activity, fluoro-cytidine metabolites with potential toxicity may accumulate more. CDA expression variation presents a relevance to polymorphism in the CDA promoter region and can affect gemcitabine and capecitabine metabolism. Studies have confirmed the relevance of variation of CDA promoter region, c.92A>G (rs602950) and c.451C>T (rss532545), to grade 2-4 diarrhea [19]. Besides, patients with CDA rs1048977-CC genotype hold a larger likelihood of adjuvant capecitabine discontinuation considering the toxicity, and capecitabine monotherapy shows a larger possibility of treatment discontinuation [25]. It has also been reported that patients carrying CDA rs1048977-T allele have a higher risk of capecitabine toxicity [26]. Variations in CDA also exhibit a certain linkage with hand-foot syndrome (HFS). Caronia et al. confirmed an obvious relevance of CDA c.-451C >T variant allele to grade 3 HFS [27]. Similarly, CES genetic variants are associated with capecitabine toxicity. In recent studies, scientists have identified novel genetic variants of carboxylesterase 1 (CES1) that may indicate severe early toxicity associated with Capecitabine [25]. In their study, the CES1P1 rs7187684-T allele could elevate the possibility of treatment discontinuation because of the toxicity, and the CES1 rs71647871-A allele could partially elevate the risk of severe HFS in capecitabine-treated patients. According to the study by Hamzic et al., in patients with solid neoplasms who received capecitabine-based treatment strategies, the CES1P1 rs7187684-T allele and the CES1P1 rs11861118 g allele could easily lead to an early onset of toxicity [28].

The TYMP gene takes charge of encoding the TP enzyme. TP greatly impacts capecitabine’s bioactivation. According to studies, the presence of TYMP rs11479-TT genotype could raise the incidence of severe nausea [25]. Existing researches fail to achieve a consensus about how TYMP rs11479 SNP affects the toxicity of fluorouracil-based therapy. The study of Jennings et al. included 254 CRC cases of Caucasian origin who had received FP treatment, confirming that carriers of the TYMP rs11479-T allele were relevant to the overall toxicity, and toxicity events-induced treatment delays (CC vs. CT/TT) [29]. Comparatively, studies by Caronia et al. that focused on 130 Spanish patients with CRC and BR who were administered capecitabine [27], Meulendijks et al. that adopted 185 Dutch-origin GC patients receiving capecitabine-based therapeutic strategies [30], and Chen et al. that included 198 GC Chinese-origin patients receiving capecitabine-based therapeutic strategies [31], did not ascertain such similar association. Obviously, Chen et al. ascertained that the carriers of the TYMP rs11479-T allele partially triggered the high risk of HFS and grade 2 anemia [31], indicative of the stronger sensitivity of the T allele carriers to capecitabine versus C allele carriers. However, the impact of this SNP on TP activity and corresponding relevance remains uncertain. All these findings necessitate a deeper understanding of the relationship between the TYMP rs11479 SNP and its effect on toxicity during capecitabine therapy. In addition, there is literature that also highlighted the obvious relevance of MTHFR C.1298A >C polymorphism to grade 2-3 HFS. From the perspective of HFS exclusively, no adverse reactions related to HFS were observed in the patient we reported. It may reflect, on the flip side, the fact that this patient may not have relevant SNPs on the capecitabine-activated pathway, while in fact we didn't test for them.

Genetic variations and polymorphisms may impact drug pharmacokinetics or pharmacodynamics and explain patients’ different responses and adverse events after treatment. One of the most commonly investigated genes in the field of fluorouracil toxicity is dihydropyrimidine dehydrogenase (DPYD). However, genotyping of these SNPs alone only occupies 30% of capecitabine therapy-related severe toxicity events [32], and the remaining toxicity is possibly caused by other genetic variants that participate in the capecitabine (CDA, CES, TYMP, TYMS, MTHFR) pharmacokinetics. Although polymorphism in the MTHFR gene is an extremely rare cause of fluoropyrimidine toxicity, our findings indicate that MTHFR (677C>T) variants may be involved in capecitabine toxicity. Therefore, this also gives us a hint that it is very important for enzyme activity testing or gene mutation testing in clinical practice, to ensure safe and efficient treatment for patients planning to receive 5-FU or capecitabine therapy. In addition to DPYD metabolic status, which is currently used in clinical practice, other genetic polymorphism screening may assist in guaranteeing the safety of adopting oral capecitabine clinically.

## Conclusions

Despite that capecitabine chemotherapy is usually well tolerated, severe and potentially fatal toxicities could occur in some patients, therefore clinicians must remain vigilant. Severe and fatal fluoropyrimidine toxicity is frequently attributed to partial or complete deficiencies in the dihydropyrimidine dehydrogenase (DPD) enzyme. It is also prudent to consider mutations in methylene tetrahydrofolate reductase (MTHFR), thymidylate synthase gene (TYMS), as well as capecitabine’s bioactivation pathway (CDA, CES, TYMP) that may contribute to such severe reactions. The study reports a case of severe toxicity in a Chinese woman with rectal cancer, attributed to a substandard dose (825mg/m2 bid) of capecitabine, possibly due to a mutation in the MTHFR gene. This case report highlights the fact that MTHFR gene polymorphism is also an important cause of capecitabine toxicity. Therefore, we think that it is significant to accumulate such case reports for further understanding. Additionally, it demonstrates how gene sequencing facilitates personalized treatment approaches and predicts potential harm associated with fluoropyrimidines. Therefore, we strongly recommend considering enzyme activity or gene mutation testing in clinical practice for patients undergoing 5-FU or capecitabine therapy to guide treatment decisions and enhance safety and efficacy.
